# The Clinical Utility of miR-21 and let-7 in Non-small Cell Lung Cancer (NSCLC). A Systematic Review and Meta-Analysis

**DOI:** 10.3389/fonc.2020.516850

**Published:** 2020-10-19

**Authors:** Cecilia Pop-Bica, Sebastian Pintea, Lorand Magdo, Roxana Cojocneanu, Diana Gulei, Manuela Ferracin, Ioana Berindan-Neagoe

**Affiliations:** ^1^Research Center for Functional Genomics, Biomedicine and Translational Medicine, “Iuliu-Hatieganu” University of Medicine and Pharmacy Cluj-Napoca, Cluj-Napoca, Romania; ^2^Department of Psychology, Babes-Bolyai University, Cluj-Napoca, Romania; ^3^Research Center for Advanced Medicine MedFuture, “Iuliu Hatieganu” University of Medicine and Pharmacy Cluj-Napoca, Cluj-Napoca, Romania; ^4^Pathology Unit, Department of Experimental, Diagnostic and Specialty Medicine, DIMES, University of Bologna, Bologna, Italy; ^5^Department of Functional Genomics and Experimental Pathology, The Oncology Institute “Prof. Dr. Ion Chiricută”, Cluj-Napoca, Romania

**Keywords:** prognostic, biomarker, NSCLC, miR-21, let-7, patients

## Abstract

Non-small cell lung cancer (NSCLC) remains a problem worldwide due to its rapid progression and low rate of response to treatment. The heterogeneity of these tumors observed in histopathology exam but also in the mutational status and gene expression pattern makes this malignancy difficult to treat in clinic. The present study investigated the effect of miR-21 and let-7 family members as prognostic biomarkers in NSCLC patients based on the results published in different studies regarding this subject until March 2019. The analysis revealed that these two transcripts are steady biomarkers for prediction of patient outcome or survival. Upregulated expression of miR-21 is associated with poor outcome of patients with NSCLC [HR = 1.87, 95% CI = (1.41, 2.47), *p* < 0.001]. The analysis regarding let-7 family, specifically let-7a/b/e/f, revealed that downregulated expression of these transcripts predicts poor outcome for NSCLC patients [HR = 2.61, 95% CI = (1.58, 4.30), *p* < 0.001]. Besides, the reliability of these microRNAs is reflected in the fact that their prognostic significance is constant given the different sample types (tissue, FFPE tissue, serum, serum/plasma or exosomes) used in the selected studies.

## Introduction

Lung cancer represents the leading cause of cancer related deaths, with more than 1.7 million deaths in 2018, being also one of the most diagnosed oncogenic pathology worldwide, alongside with breast cancer (1). Non-small cell lung cancer (NSCLC) accounts for more than 85% of the lung cancers, having two major histological types—adenocarcinoma and squamous cell carcinoma (2). According to the reports of World Health Organization (WHO), smoking habits in both sexes appear to indicate a decrease, from 22.1% in 2010 to an expected 17.3% in 2025 (3). Nevertheless, lung cancer incidence in patients increased, raising serious concerns in terms of prevention and response to therapy (1). The explanation would be that, independent to exogenous carcinogens, lung tumorigenesis is driven by genetic dysregulations of coding and non-codingRNAs (4, 5).

Understanding the molecular imbalance that underlies a malignancy is the key to develop and apply an efficient therapy scheme (6). In NSCLC, precisely adenocarcinoma, there are several targeted therapies developed to overcome the disequilibrium that leads to the transformation and abnormal behavior of cells. Mutations in epidermal growth factor receptor (EGFR), or other recombinant DNA processes in anaplastic lymphoma kinase (ALK) or c-ros oncogene 1 (ROS1) represent the main alterations that occur in patients diagnosed with NSCLC (7). Another important determinant in the development of the malignant phenotype in NSCLCs is represented by the non-coding part of the genome (8). Over the last two decades, microRNAs (miRNAs) gained the attention of the medical researchers, as they were proved to be important players in cancer development (9–11). MiRNAs represent short RNAs that do not undergo the translation into proteins, but regulate the expression of many protein coding genes, by complementary binding (12). Considering the dysregulation of miRNAs, these molecules can be categorized as tumor suppressor or oncogenic miRNAs (13, 14).

Intensively studied let-7 family is known to have regulatory function in several cancers, such as pancreatic cancer (15), thyroid cancer (16), and colorectal cancer (17), with differences in the expression profile of different members of let-7 family. In thyroid cancer, let-7b appears to be downregulated, while let-7f is overexpressed in tissue samples collected from patients with follicular thyroid cancer (16). Target genes of let-7 include c-Myc (18), high-mobility group A (HMGA) (19–21) or signal transducer and activator of transcription 3 (STAT3) and Janus-activated kinase 2 (JAK2) (15), genes involved in cell proliferation and cell cycle. In NSCLC cells, let-7 family transfection considerably reduced proliferation rate, hence acting as tumor suppressor gene (22). By regulating the Ras family, let-7 takes part in controlling cellular proliferation in *in vivo* models, although treatment with this miRNA revealed that there are additional mechanisms with downstream activation that lead to resistance of cancer cells to the tumor suppressive functions of this transcript (23). In NSCLC, let-7 underexpression was reported as significantly correlated with patient outcome (24–26), but there are studies that were not successful in establishing a direct link between low expression of let-7 and prognostic of NSCLC patients (27, 28).

The oncogenic role of miR-21 in patients with NSCLC was reported in a substantial number of publications (24–27, 29–47). MiR-21 appears to be upregulated in many malignancies, such as breast cancer, head and neck cancers, non-small cell lung cancer, melanoma, glioblastoma, confirming its oncogenic role. Its function is exerted by downregulating the expression of suppressor of cytokine signaling 1 and 6 (SOCS1/6) and phosphatase and tensin homolog (PTEN), thus distorting the apoptotic mechanism, cellular growth and proliferation of NSCLC cells (48–50). Further information regarding the involvement of miR-21 in different cellular processes and pathways in cell lines/animal models and in NSCLC patients' outcome is systematically formulated in a previous review (51).

Given the differences of the results obtained by various groups regarding both miR-21 and let-7, the focus of this study was to evaluate the prognostic significance of the expression levels of miR-21 (as oncogene) and let-7 (as tumor suppressor gene) in patients with NSCLC. Therefore, a comprehensive literature search was assessed to perform a meta-analysis on these two molecules and their implication in patient outcome.

## Methods

### Search Procedure and Study Selection

In order to perform this meta-analysis, the PRISMA (Preferred Reporting Items for Systematic Reviews and Meta-Analyses) protocol was followed (52, 53). The comprehensive search for eligible studies began by interrogating the PubMed database, as it provided the literature most consistent with the topic. The database was scrutinized using the following terms: “let-7^*^” OR “let7^*^” AND “lung cancer” for let-7 family, and “miR-21” OR “microRNA 21” AND “lung cancer” AND “patients” for miR-21. The searching terms for let-7 family were simplified due to the poor results generated using the same search scheme that was applied for miR-21 in lung cancer.

The articles published until 20 March 2019 were assessed for eligibility, reviewing the titles and abstracts, and full-text publications where the abstract text was inconclusive. Publications listed that were written in another language than English, review/meta-analysis/letters/erratum papers were all ruled out from the meta-analysis. Publications that did not assess the association between the expression levels of miR-21/let-7 with the survival in patients with lung cancer, specifically NSCLC, were not selected for the analysis.

Publications had several inclusion criteria, as follows: (i) studies that characterized patients with NSCLC, including sample size, sample type, and histological type; (ii) miR-21/let-7 evaluation of expression in human samples; (iii) Hazard ratios (HR) calculated in relation with specific microRNA expression for overall survival (OS), progression-free survival (PFS), or recurrence free survival (RFS), along with the *p*-values and 95% confidence intervals (95% CIs).

Each set of publications (for miR-21 and let-7) was evaluated and verified by two independent reviewers (C.P-B. and L.M). Publications that included any data regarding the investigation of the correlation between the expression levels of the selected microRNAs and survival time of patients suffering from NSCLC were organized in an excel worksheet.

The following parameters were marked from the eligible studies for this meta-analysis: (i) reference data (authors, year); (ii) demographic characteristics: population, male/female ratio, ethnicity, smoking status; (iii) pathology data: histology, stage, TNM status; (iv) experiment design: sample type, techniques, microRNA expression; (v) statistical data: survival type, hazard ratio (HR), 95% confidence interval (CI), *p*-value, statistical tests/methods.

### Data Set and Coding Procedure

In order to standardize the way results were reported across studies and compare survival between miR-21 and let-7, we applied the following procedure. All hazard ratios (HR) reported for miR-21 were computed and introduced in our database as high/low expression, while for let-7 results were computed as low/high. As a consequence, for miR-21, HR > 1 indicates higher mortality for high expression, while for let-7 HR > 1 indicates higher mortality for low expression.

### Data Analysis

Analyses were conducted by using Comprehensive Meta-Analysis software, version 2.2.050 (Biostat Inc., Englewood, NJ, USA). As an indicator of effect sizes, the hazard ratio was used. Given the heterogeneity of the studies, all analyses were based on a random effects model.

### Publication Bias Analysis

To assess the risk of publication bias for the results we calculated the Begg and Mazumdar's rank correlation test according to the recommendations of Kepes et al. (2012) (54). This test computes the rank order correlation (Kendall's tau b) between the effect size and the standard error (which is driven primarily by sample size). This test identifies if large studies tend to be included in the analysis regardless of their effect size, whereas small studies are more likely to be included when they show a relatively large effect size.

### TCGA Analysis

Data analysis was also performed using The Cancer Genome Atlas (TCGA) data available for lung adenocarcinoma (LUAD) and lung squamous cell carcinoma (LUSC) and downloaded from FireBrowse as one data matrix including the expression levels of the mature miRNA as normalized and log2 transformed values. Clinical data and follow-up data for these patients were downloaded from the UCSC (University of California Santa Cruz) Xena browser, and then matched with the expression data. Kaplan-Meier survival analysis was carried out only on samples which included complete survival data. Therefore, the patients were separated based on the miRNA expression levels as high-expression and low-expression according to the median value in the group. This analysis was initially performed on the entire cohort from the TCGA set, and then the cohort was split into males and females, and the same investigation was performed.

## Results

### Qualified Studies

**Figure 1:**
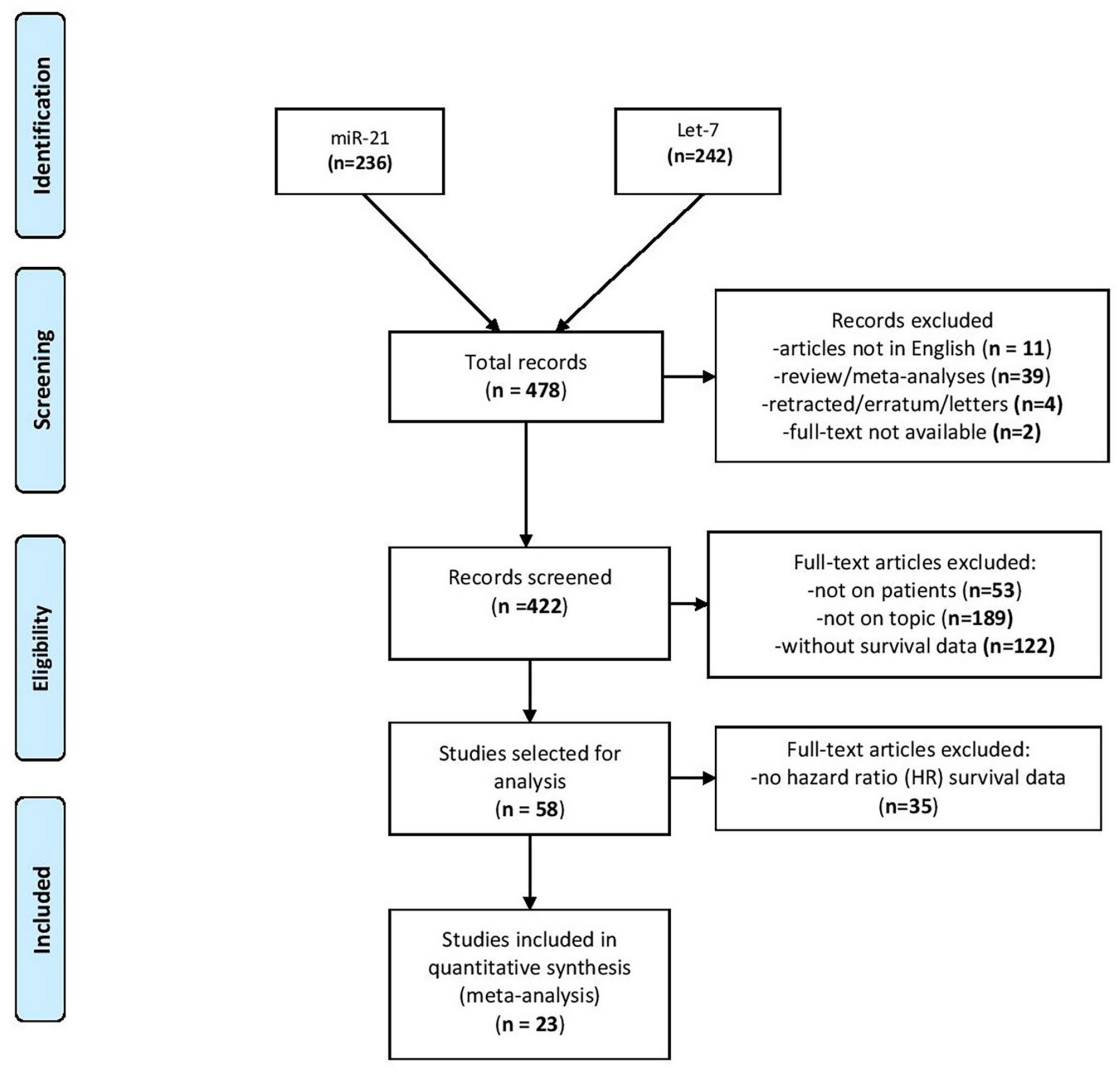
Flowchart of selection of articles and exclusion criteria.

After interrogating the PubMed database, the search disclosed 236 publications for miR-21 and 242 publications for let-7 family (from now on reported for simplicity as “let-7”) for lung cancer topics. From the total number of 478 articles revealed after the search, 56 publications were expelled as by the criteria presented in the Methods section. The abstracts and full-text articles of 422 studies were selected for a more exhaustive assessment. Another 364 studies were excluded as they were not on topic or the expression of miRNAs was not evaluated in patients or was not precisely linked to the effect on patient outcome. Therefore, 58 articles were selected as candidate studies for further analysis. From these, 35 studies did not provide HR data in the survival analysis or the effect of miR-21/let-7 on patient outcome was not evaluated alone. Finally, 23 articles were identified as eligible for the present meta-analysis (24–27, 29–47), of which two studies provided HR survival data for both miR-21 and let-7 (Figure 1) (27, 32).

### Study Characteristics

From the 23 studies selected for the meta-analysis, sample size ranged between 10 and 638 patients, with a median study size of 81.5, categorized according to ethnicity: Chinese, Bosnian and Serbian, Greek, Japanese, Italian, and mixed population. The studies used as sample fresh frozen tissue (26, 29, 30, 32–34, 37, 43–46), formalin-fixed paraffin embedded (FFPE) tissue (24, 25, 27, 31, 35, 38, 40), serum (30, 36, 39, 41, 43) or plasma exosomes (42, 47) (two studies used both tissue and serum) (30, 43). For miRNA quantification, microarray and qRT-PCR were used in six studies (30, 31, 43, 44, 46, 47), *in situ* hybridization (ISH) and qRT-PCR or Nanostring were used in one study, and in rest of the studies quantitative reverse-transcription polymerase chain reaction (qRT-PCR) was mainly used. The prognostic significance of miR-21/let-7 was reported in all 23 studies listed in Table 1.

**Table 1:** Main features and results of the 23 selected studies.

**First author**	**Year**	**Ethnicity**	**Sample type**	**N**	**miRNA**	**Expression**	**miRNA assay**	**Survival**	**HR**	**95%CI**	***p*-value**	**Normalization**
Yanaihara	2006	American (mixed)	Tissue	52	let-7a	↓	qRT-PCR	CSS	2.35	1.08–6.86	0.033	Normalization to median array
Takamizawa	2004	Japanese	Tissue	143	let-7a/f	↓	qRT-PCR	Post-operative survival	2.17	1.21–3.89	0.009	5S rRNA
Zhu	2014	Chinese	Tissue	70	let-7e	↓	Microarray/qRT-PCR	OS	0.058	0.010–0.334	0.001	U6 snRNA and U48 snRNA
			Serum					OS	0.686	0.220–2.135	0.515	
Zhang	2012	Chinese	FFPE tissue	54	let-7e	↓	Microarray/qRT-PCR	CSS	1.04	1.01–1.07	0.004	U6 snRNA and U48 snRNA
		Chinese	FFPE tissue	57	let-7e	↓	Microarray/qRT-PCR	CSS	9.04	2.55–32.05	0.001	
Jusufovic	2012	Bosniak and serbian	FFPE tissue	10	let-7b	↓	qRT-PCR	PFS	0.14	0.07–0.29	<0.0001	RNU6B
Landi	2010	Mixed	FFPE tissue	290	let-7e	↓	Chip array/qRT-PCR	OS	0.46	0.27–0.8	0.006	RNU6B
Voortman	2010	Mixed	FFPE tissue	638	let-7a		qRT-PCR/ISH	OS	0.84	0.68–1.04	0.11	RNU66
				631	miR-21		qRT-PCR/ISH	OS	0.81	0.650–1.01	0.06	
Markou	2008	Greek	Tissue	48	miR-21	↑	qRT-PCR	OS	2.533	1.066–6.02	0.035	U6
Saito	2011	Mixed	Tissue	126	miR-21	↑	qRT-PCR	CSM	2.25	1.32–3.82	0.003	RNU66
		Japanese	Tissue	191	miR-21	↑	qRT-PCR	RFS	2.66	1.47–4.83	0.001	
Wang	2017	Chinese	FFPE tissue	216	miR-21	↑	qRT-PCR	survival	1.002	1.001–1.003	0.007	U6
Wang	2011	Chinese	Serum	88	miR-21	↑	qRT-PCR	OS	2.01(RR)	1.78–3.26	0.015	U6
Wang	2013	Chinese	Tissue	60	miR-21	↑	qRT-PCR	Post-operative survival	2.103	0.695–3.078	0.032	U6B
Ulivi	2019	Italian	Serum	83	miR-21–5p		qRT-PCR	DFS	0.66	0.44–0.97	0.035	miR-221-3p and miR-24-3p for SCC; miR-221-3p and miR-126-3p for ADC; and cel-miR-39
Kunita	2018	Japanese	FFPE tissue	89	miR-21-5p	↑	ISH	Survival	5.494	1.431–18.17	0.0158	U6
Le	2012	Chinese	Serum	82	miR-21	↑	qRT-PCR	OS(pre-op)	2.664	0.949–7.478	0.063	miR-16
								OS (post-op)	1.362	0.447–4.153	0.587	
Li	2017	Chinese	Tissue	78	miR-21	↑	qRT-PCR	OS	1.755	0.63–4.886	0.282	U6
		Chinese	Tissue	78	let-7a		qRT-PCR	OS	0.332	0.068–1.614	0.172	
Liu	2017	Chinese	Plasma exosomes	196	miR-21-5p	↑	qRT-PCR	OS	2.12	1.28–3.49	0.003	let-7a-5p
Liu	2012	Chinese	Tissue	70	miR-21	↑	qRT-PCR/microarray	OS	3.187	0.368–27.592	0.123	U6
		Chinese	Serum	70	miR-21	↑	qRT-PCR/microarray	OS	4.316	1.265–19.206	0.046	
Gao	2012	Chinese	Tissue	58	mir-21	↑	qRT-PCR/microarray	DFS	2.82	1.091–7.285	0.032	RNU6B
Akagi	2013	Japanese	Tissue	199	miR-21	↑	Nanostring+qRT-PCR	DFS	1.34	0.67–2.69	0.410	Average expression of miR-720, miR- 26a, miR-126, miR-16, and miR-29a(nanostrig) Absolute quantification (qRT-PCR)
		Mixed	Tissue	89	miR-21	↑	Nanostring+qRT-PCR	DFS	3.42	1.66–7.04	0.001	
		Japanese	Tissue	136	miR-21	↑	Nanostring+qRT-PCR	DFS	1.73	0.60–4.98	0.312	
		Mixed	Tissue	47	miR-21	↑	Nanostring+qRT-PCR	DFS	6.55	1.97–21.78	0.002	
Gao	2011	Chinese	Tissue	30	miR-21	↑	qRT-PCR/microarray	survival	1.293	1.123–1.489	0.000	RNU6B
Dejima	2017	Japanese	Plasma exosomes	195	miR-21	↑	qRT-PCR/microarray	DFS	3.81	1.13–14.93	0.018	miR-16
Xu	2018	Chinese	FFPE tissue	111	miR-21-5p	↑	qRT-PCR	PFS	2.164	1.406–3.332	<0.001	U6

*CSS, cancer-specific survival; DFS, disease-specific survival; OS, overall survival; PFS, progression-free survival; RFS, relapse-free survival*.

### Prognostic Significance of miR-21/let-7 in NSCLC

All 23 selected studies reported a direct connection between the expression levels of miR-21/let-7 and the outcome of lung cancer patients. The outcome is reported in these publications as: overall survival (OS), progression-free survival (PFS), relapse-free survival (RFS), disease-free survival (DFS), cancer-specific survival (CSS), post-operative survival or survival.

#### Overall HR for miR-21

**Figure 2:**
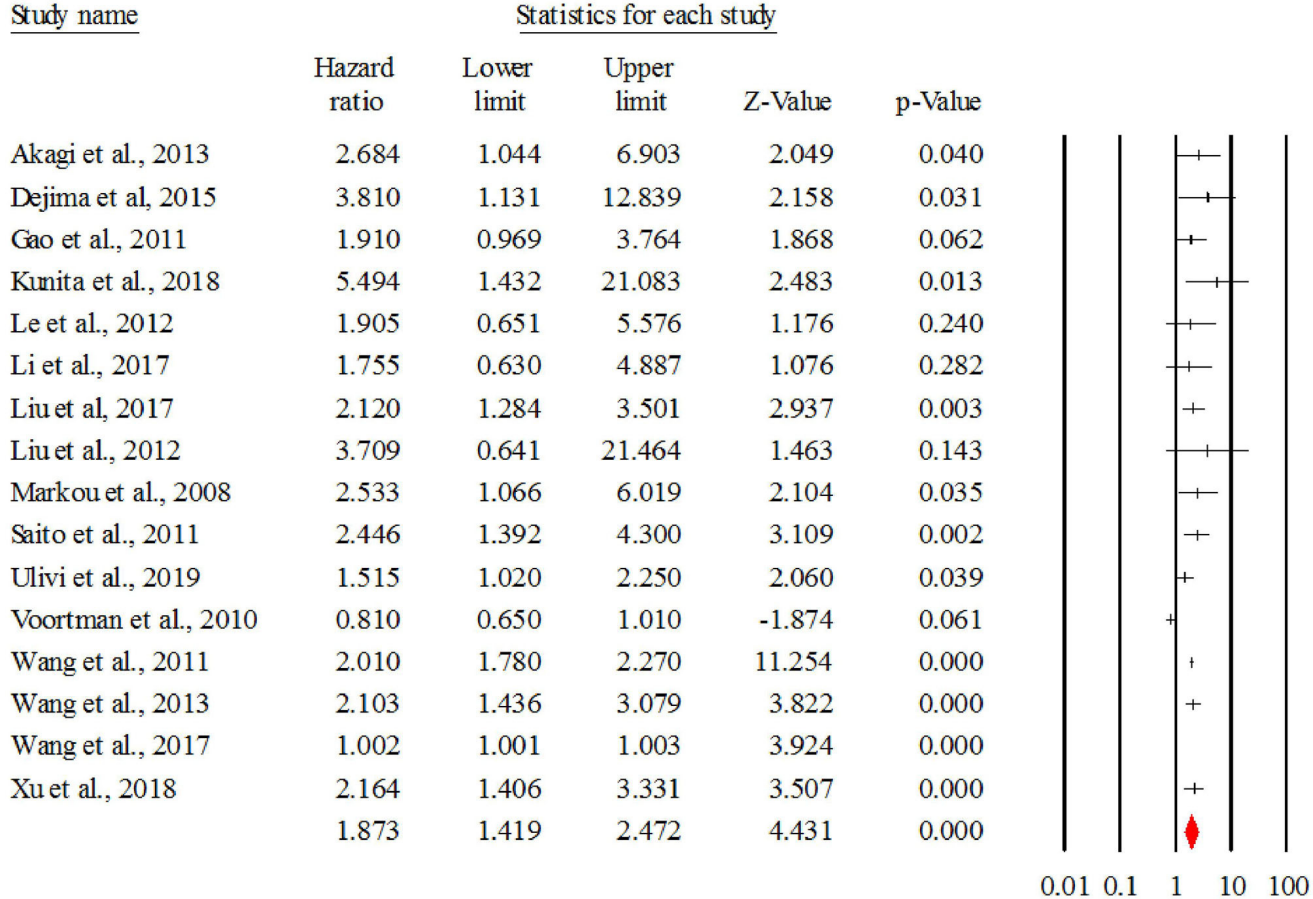
The forest plot of HR from all studies assessing the prognostic significance of miR-21 in NSCLC patients and summary.

Figure 2 describes the forest plot for miR-21 with the hazard ratio for each study included in the meta-analysis. The overall corrected hazard ratio for miR-21 is HR = 1.87, 95% CI = (1.41, 2.47) and statistically significant (*Z* = 4.43, *p* < 0.001), indicating that a high expression of miR-21 is associated with poor survival.

**Figure 3:**
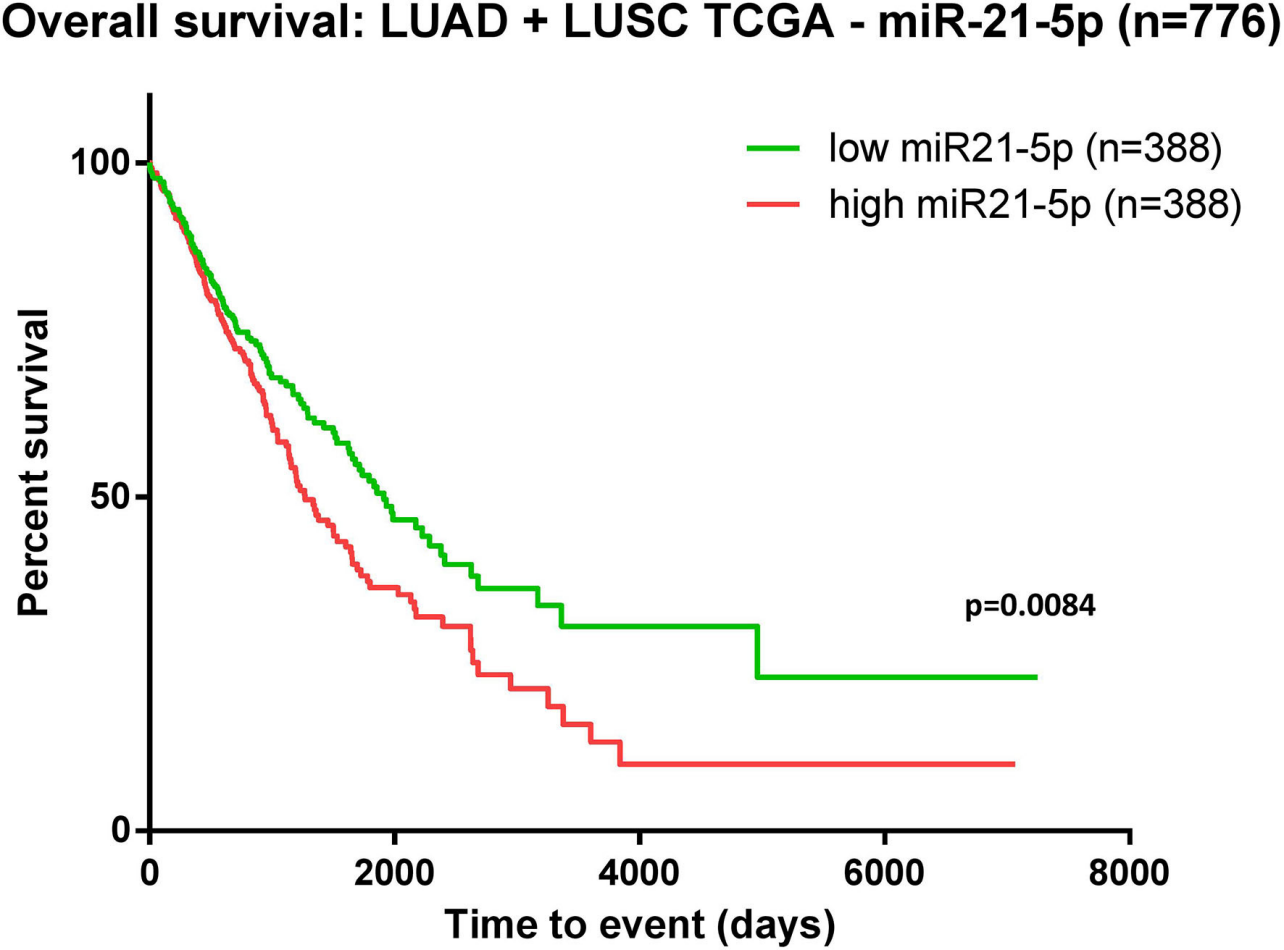
Kaplan-Meier curve using TCGA data analysis regarding the expression of miR-21 in patients diagnosed with lung adenocarcinoma (LUAD) and lung squamous cell carcinoma (LUSC) (*n* = 776). Patients were classified as high-/low-expression according to the median value. Upregulated expression of miR-21 is correlated with poor survival (*p* = 0.0084).

The data downloaded from TCGA revealed 776 patients with LUAD/LUSC with complete clinical and survival data available. Kaplan-Meier survival analysis was performed on these samples organized as high-expression and low-expression compared to the median value. The evaluation revealed that overexpression of miR-21 in NSCLC patients was associated with poor overall survival (*p* = 0.008) (Figure 3).

#### Overall HR for let-7

**Figure 4:**
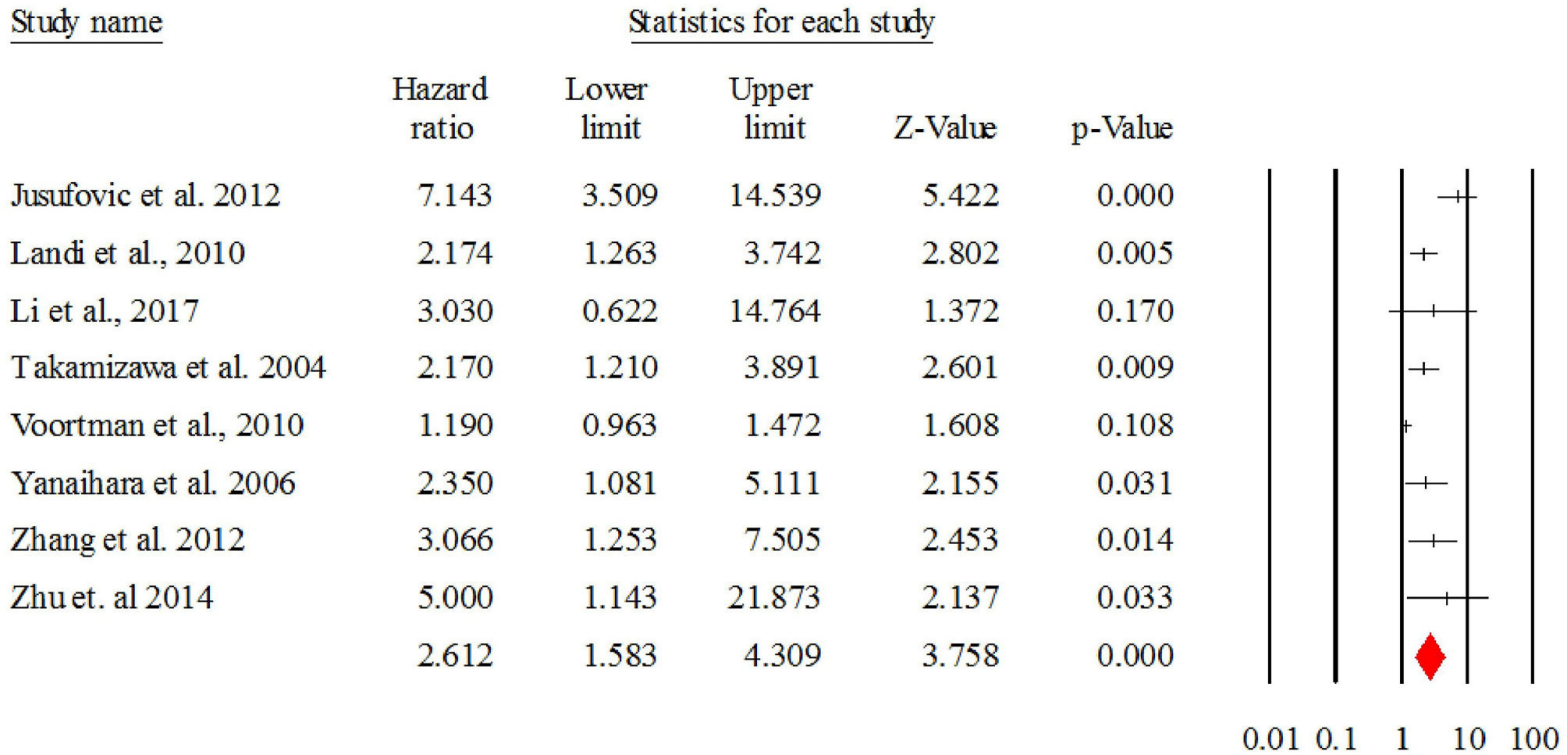
The forest plot of HR from all studies assessing the prognostic significance of let-7 in NSCLC patients and summary.

Figure 4 describes the forest plot for let-7 with the hazard ratio for each study included in the meta-analysis. The overall corrected hazard ratio for let-7 is HR = 2.61, 95% CI = (1.58, 4.30) and statistically significant (*Z* = 3.75, *p* < 0.001), indicating that a low expression of let-7 is associated with poor survival.

#### MiR-21 vs. let-7

The comparison between the hazard ratios generated by miR-21 (high vs. low) vs. let-7 (low vs. high) indicated that there were no significant differences, Q(1) = 1.30, *p* = 0.253.

### Publication Bias Analysis

For miR-21, the publication bias analysis using the Begg and Mazumdar's rank correlation test revealed a non-significant Kendall's tau b of −0.14, with a two-tailed *p*-value of 0.444 (based on continuity-corrected normal approximation), suggesting there is no tendency for studies which are more precise (and implicitly larger) to generate larger effect sizes.

The same analysis performed for let-7 revealed a non-significant Kendall's tau b of 0.10, with a two-tailed *p*-value of 0.710 (based on continuity-corrected normal approximation), suggesting there is no tendency for studies which are more precise (and implicitly larger) to generate larger effect sizes.

### Heterogeneity Analysis

For miR-21, the distribution of effects proved to be significantly heterogeneous, Q(15) = 206.17, *p* < 0.001, leading us to perform the moderators' analysis to test several explanations for this heterogeneity.

In the case of let-7, the distribution of effects proved to be non-significantly heterogeneous, Q(7) = 32.58, *p* < 0.001. As a consequence, we performed the moderators' analysis to test several explanations for this heterogeneity.

Also, because of this heterogeneity, all analysis were performed under the random effects model.

### Moderators' Analysis for miR-21

#### Categorical Moderators

##### Population

As Table 2 shows, the hazard ratios obtained by studies performed on Chinese and Japanese population proved to be statistically significant, HR = 1.85, 95% CI = (1.25, 2.73), *p* = 0.002 for Chinese samples, and HR = 2.78, 95% CI = (1.39, 5.55), *p* = 0.004 for Japanese samples. The hazard ratio obtained on Greek and Italian population seems to be not significant, but these results should be interpreted with caution since they are represented in our meta-analysis by only one study each.

**Table 2:** The analysis of HR (miR-21) as a function of population, sample type, and existence of pre-treatment.

**Moderator**	**Categories**	**No of studies**	**HR**	**Inf (95% CI)**	**Sup (95% CI)**	***Z***	***p***	***Q***	**df**	***p***
**Population**										
	Chinese	9	1.85	1.25	2.73	3.08	0.002	1.60	4	0.809
	Greek	1	2.53	0.68	9.39	1.39	0.164			
	Italian	1	1.52	0.53	4.37	0.76	0.442			
	Japanese	4	2.78	1.39	5.55	2.90	0.004			
	Mixed	3	1.71	0.89	3.32	1.60	0.109			
**Sample type**										
	Liquid biopsy	6	2.10	1.43	3.09	3.77	0.000	0.72	1	0.394
	Tissue	11	1.70	1.28	2.26	3.70	0.000			
**Pre-treatment**										
	No	11	2.13	1.46	3.10	3.97	0.000	0.11	1	0.730
	Yes	4	1.90	1.11	3.25	2.34	0.019			

##### Sample type

The analysis of hazard rations as a function of sample type revealed significant HR for both type of samples analyzed, HR = 2.10, 95% CI = (1.43, 3.09), *p* < 0.001 for liquid biopsy and HR = 1.70, 95% CI = (1.28, 2.26), with no significant differences between those two categories, Q(1) = 0.72, *p* = 0.394.

##### Pre-treatment

Our analysis indicated that the existence of pre-treatment did not make any difference regarding the hazard ratio obtained, Q(1) = 0.11, *p* = 0.730, both categories of studies (with *vs*. without pre-treatment) yielding significant hazard ratios.

#### Continuous Moderators

We used percentage of women in the samples, percentage of smokers, and percentage of patients with lymph node metastasis. In order to test continuous moderators, we performed a meta-regression in which percentage (%) of women, percentage of smokers and percentage of patients with lymph node metastasis (LNM) were treated as predictors of hazard ratios.

##### Percentage of women

Among the studies analyzed, 14 reported the proportion of women. We performed a meta-regression analysis in order to test the predictive value of the proportion of women in the samples upon the hazard ratios. The results revealed that this variable had a significant positive predictive value, *B* = 0.016, *p* < 0.001. In other words, larger proportions of women in the samples are associated with higher levels of hazard ratios obtained.

**Figure 5:**
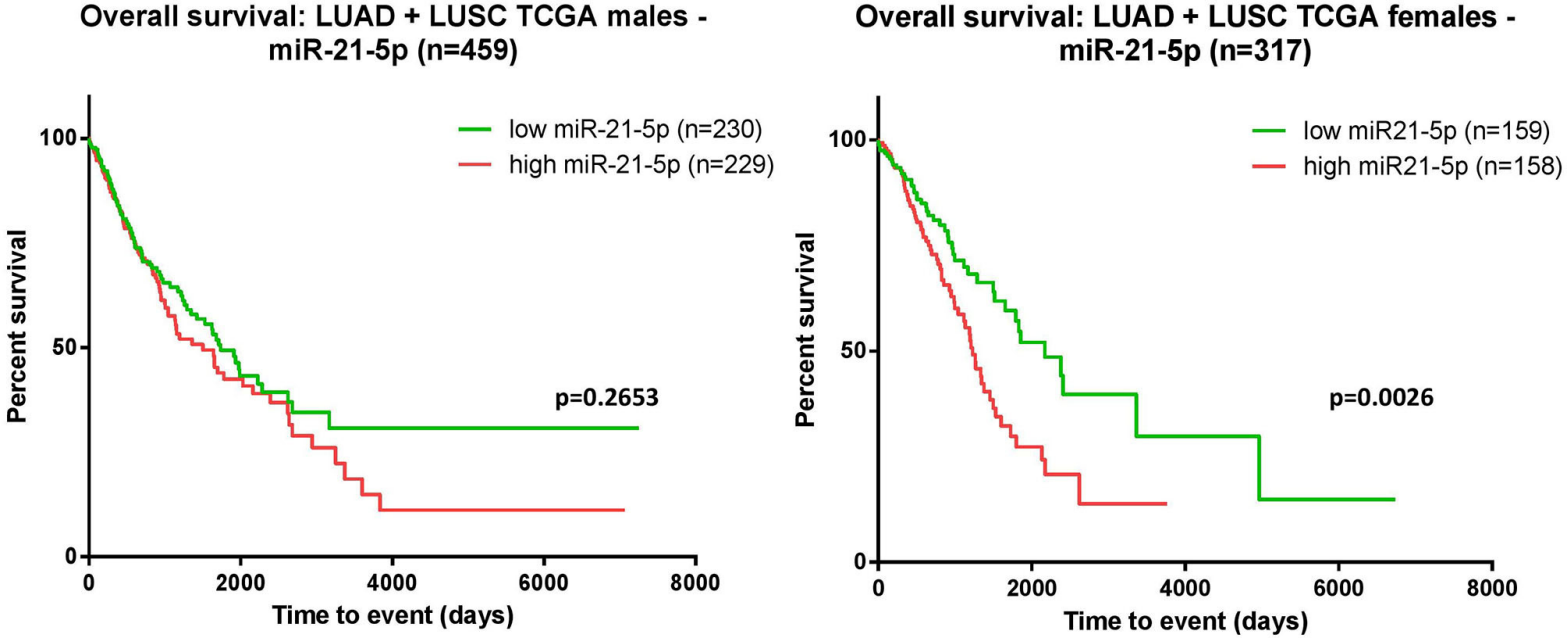
Kaplan-Meier curves. The survival analysis was assessed for the male cohort indicated that the overexpression of miR-21 and poor survival did not reach a statistic significance *p* = 0.2653 **(left)**; in females, overexpression of miR-21 correlated with poor survival *p* = 0.0026 **(right)**.

Using the TCGA data available, we performed the survival analysis separating the patients according to their gender. Consistent with the above-mentioned findings, the separate analysis—on women and men—revealed that, the correlation between overexpression of miR-21 and worse outcome is statistically significant in females, but not in males (Figure 5).

##### Percentage of smokers

Among the selected studies, only 12 reported the proportion of smokers. The analysis of the predictive value of the proportion of smokers in each sample revealed that this variable had a significant negative predictive value, *B* = −0.009, *p* = 0.002, which means that as the proportion of smokers increases, the hazard ratio decreases (gets closer to 1).

##### Percentage of patients with LNM

The meta-analytical regression performed on the 10 studies which reported the percentage of patients with LNM, proved that this percentage is a significant negative predictor of the hazard ratio, *B* = −0.017, *p* < 0.001. In other words, as the percentage of patients with LNM increases, the hazard ratio decreases (gets closer to 1).

### Moderators' Analysis for let-7

#### Categorical Moderators

We used population and pre-treatment as moderators. The type of sample (liquid biopsy vs. tissue) could not be tested as potential moderator because all studies used tissue as sample for analysis.

##### Population

As Table 3 shows, the hazard ratios obtained by studies performed on Chinese and Bosnian/Serbian populations proved to be statistically significant, HR = 2.86, 95% CI = (1.23, 6.65), *p* = 0.014 for Chinese samples, and HR = 7.14, 95% CI = (1.38, 36.97), *p* = 0.019 for Bosnian/Serbian population. The last result should be taken with reserve since it has been obtained by analyzing only one study. The hazard ratios obtained on American, Japanese and mixed populations seem to be not significant, but these results should be interpreted with caution due to the availability of one or two studies.

**Table 3:** The analysis of HR (let-7) as a function of population and existence of pre-treatment.

**Moderator**	**Categories**	**No of studies**	**HR**	**Inf (95% CI)**	**Sup (95% CI)**	***Z***	***p***	***Q***	**df**	***p***
**Population**										
	American	1	2.35	0.44	12.53	1.00	0.317	2.36	4	0.669
	Bosnian and Serbian	1	7.14	1.38	36.97	2.34	0.019			
	Chinese	5	2.86	1.23	6.65	2.45	0.014			
	Japanese	1	2.17	0.44	10.68	0.95	0.341			
	Mixed	2	1.58	0.53	4.69	0.83	0.407			
**Pre-treatment**										
	No	2	3.96	1.35	11.66	2.50	0.012	0.92	1	0.336
	Yes	1	2.17	1.21	3.89	2.60	0.009			

##### Pre-treatment

Our analysis indicated that the existence of pre-treatment did not make any difference regarding the hazard ratio obtained, Q(1) = 0.92, *p* = 0.336, both categories of studies (with vs. without pre-treatment) yielding significant hazard ratios. Being performed on a small number of studies, this analysis should be also interpreted with precaution.

#### Continuous Moderators

We used percentage of women in the samples, percentage of smokers, and percentage of patients with lymph node metastasis.

##### Percentage of women

Among the studies analyzed, eight reported the proportion of women. We performed a meta-regression analysis in order to test the predictive value of the proportion of women in the samples upon the hazard ratios. The results revealed that this variable had a significant positive predictive value, *B* = 0.014, *p* = 0.008. In other words, larger proportions of women in the samples are associated with higher levels of hazard ratios obtained.

##### Percentage of smokers

Among the selected studies, only three reported the proportion of smokers. The analysis of the predictive value of the proportion of smokers in each sample revealed that this variable had a non-significant predictive value, *B* = 0.024, *p* = 0.115. Being performed on a small number of studies, this analysis should be regarded with discretion.

##### Percentage of patients with LNM

The meta-analytical regression performed on the four studies which reported the percentage of patients with LNM, proved that this percentage is a significant negative predictor of the hazard ratio, *B* = −0.037, *p* = 0.009. In other words, as the percentage of patients with LNM increases, the hazard ratio decreases (gets closer to 1).

## Discussion

Since the recognition of miRNAs as relevant players in cancer development and progression, many studies reported the aberrant expression (overexpression or underexpression) of these RNAs in several cancers (55). This meta-analysis reports that the expression levels of both miR-21 and let-7 family are highly correlated with the outcome of NSCLC patients.

The analysis for miR-21 revealed that overexpression of miR-21 in NSCLC patients resulted in shortened survival time (*p* < 0.001), bringing new evidence of the oncogenic nature of this miRNA in lung cancer. In accordance with this result, TCGA data analysis on patients with lung adenocarcinoma (LUAD) and lung squamous cell carcinoma (LUSC) indicated a significant association between upregulated levels of miR-21 and poor survival (Figure 3). The moderators' analysis on miR-21 disclosed the evidence that regardless of sample type (liquid biopsy or tissue) the analysis showed no significant differences in HR for each category. This observation supports the idea that the abnormal upregulation of miR-21 in tumor tissue sample is also mirrored in plasma/serum samples of these patients. This consideration is not necessary valid for other cancers or other miRNAs, as there are recent publications with examples of molecules reported up-/downregulated in tissue, and opposite regulation is observed in liquid biopsies of the same patient (56, 57). One important highlight is that the pre-treatment does not affect the HRs, suggesting that in this particular case, miR-21 could be considered a stable biomarker for the prognosis of NSCLC patients. When analyzing how the proportion of women is reflected in the HRs obtained, the results showed that the accuracy of the HR calculated as high vs. low is higher when the proportion of woman increases. This means that in woman, the aberrant expression of miR-21 seems to be more consistent with its impact on patient survival. This result is also emphasized in the TCGA survival evaluation, as the survival analysis on LUAD and LUSC patients revealed that upregulated expression of miR-21 indicated a significant correlation with worse survival in female patients, but not in males (Figure 5). This result is not completely new, in a previous review published by our group, the prognostic value of miR-21 in LUAD patients from TCGA was already assessed, with the confirmation that upregulation of this transcript predicts poor outcome of LUAD patients (51) and supported by other groups' research in NSCLC TCGA datasets (58).

Another moderator investigated in this meta-analysis was smoking status. The analysis disclosed that the HR tends to get closer to 1, meaning that the differences between the outcomes of patients do not differ substantially with the proportion of smokers in the analyzed sample. This observation is not unexpected, as the effects of smoking are already notorious in prompting lung cancer (59), and the survival rates in smoking patients tend to be similar, despite their categorization as high or low expressing miR-21. One similar impact appears to have the presence of lymph node metastasis in NSCLC patients, as previously reported (60). Our results imply that patients with LNM tend to have comparable survival rates and the differences in survival rates decrease when LNM are present. All these findings, specifically the fact that the prognostic value of miR-21 appears to be more accurate in patients who are female, non-smoking or Asian, are consistent with previous studies regarding biomarkers in NSCLC patients (61, 62). Our results suggest the fact that in the mentioned categories miR-21 expression is a valuable prognosis biomarker in NSCLC patients and that in males, smokers and patients with LNM, the examination of other biomarkers should be taken into consideration when assessing the prognostic of a patient.

Analysis of the impact of let-7 family members in patients diagnosed with NSCLC indicates that low expression of this miRNA is a predictor for shortened survival rates. The inquiry comprised eight studies and the summary of the HR is statistically significant, emphasizing the importance of let-7's expression in NSCLC. The TCGA data analysis did not confirm this finding (Supplementary Material), although this is not unexpected, given the fact that although the members of let-7 family possess similar functions in humans (63), there are definite differences between the representatives of this family and their role in mediating particular aspects of malignant development and progression. As the TCGA survival analysis performed separately on LUAD and LUSC datasets for the transcripts (miR-21, let-7a/b/e/f) indicated (Supplementary Figure 1), in LUAD, the expression of these microRNAs appears to have a significant correlation with the outcome of these patients. This may be regarded as a consequence of the fact that even though the two classes of cancers—lung adenocarcinoma and squamous cell carcinoma—are part of the same pathology—NSCLC-, the differences in the genomic mutational landscape have consequences observed at molecular level. In this respect, for LUAD patients' mutations in TP53, STK11, ALK, EGFR genes are more common, unlike LUSC patients, which have more characteristic mutations in RB1, KEAP, NFE2L2 or NF1 genes (64–68). The literature mentions the efficacy of let-7b in NSCLC cell lines with TP53 and EGFR mutations as an adjuvant in the therapy with erlotinib in order to potentiate the effect of this therapeutic compound (69). Besides, In NSCLC miR-21 was reported as being regulated by EGFR in non-smoker patients, its upregulation being correlated with EGFR mutations (70). Given the fact that EGFR and TP53 are genes more commonly mutated in LUAD patients, this would bring an explanation regarding the results of the TCGA survival analysis. When considering pre-treatment in these patients, the examination indicated that HR is not influenced by a previous chemo-/radiotherapy. Given the small number of studies in which treatment information was available or patients had undergone treatment before sample collection, this aspect should be further investigated for solid evidences. As was the case for miR-21, the percentage of women positively influenced the HR, suggesting that the expression profile of let-7's as survival predictor is more stable in women. As expected, the presence of LNM appears as a negative predictor for HR, the survival rates of high/low expressing let-7 patients tend to be similar in patients who already developed LNM, as the spreading of the tumor cells—leading to the development of new tumors—is harder to control and eventually leads to decease. One interesting observation is that smoking does not affect HRs and smoking patient's survival rates differ according to their high/low expression levels of let-7.This observation has to be confirmed in additional studies, as the number of present studies reporting the smoking status of the patients is rather small (three studies). As in miR-21, expression levels of let-7 have a more accurate prognostic value in females, rather than in males and patients presenting LNM. The present meta-analysis proved that miR-21 and let-7 can serve as prognostic biomarkers in NSCLC patients, although there are several drawbacks that need to be addressed. Since smokers represent an important part of patients with NSCLC, it would argue against the fact that these microRNAs alone could be used as prognostic biomarkers in NSCLC. There is a clear need to further investigate other molecules or expression patterns in a group of molecules in order to establish a definite prognostic biomarker/panel in smokers. Besides, in the absence of consistency among studies regarding patient characteristics, clinical or methodological settings, the constructive heterogeneity hindered us the possibility to investigate the influence of other moderators on miR-21 and let-7 stability as prognostic biomarkers in NSCLC patients. In order to reinforce the importance and the utility of these two transcripts in non-smoker NSCLC patients, future studies focused exclusively on non-smoker patients should be conducted to investigate their roles and/or correlation with common mutations encountered also in non-smoking patients diagnosed with NSCLC, such as EGFR, ALK, KRAS, RET, etc. (71).In this respect, a meta-analysis published in 2016 revealed that in the genes most frequently mutated in NSCLC, such as EGFR, KRAS, and ALK mutations are associated with patient characteristics, such gender, smoking status and histological type (72). As already stated, in non-smokers, miR-21 appears to be regulated by *EGFR*, miR-21 overexpression being associated with *EGFR* mutations (70).

**Figure 6:**
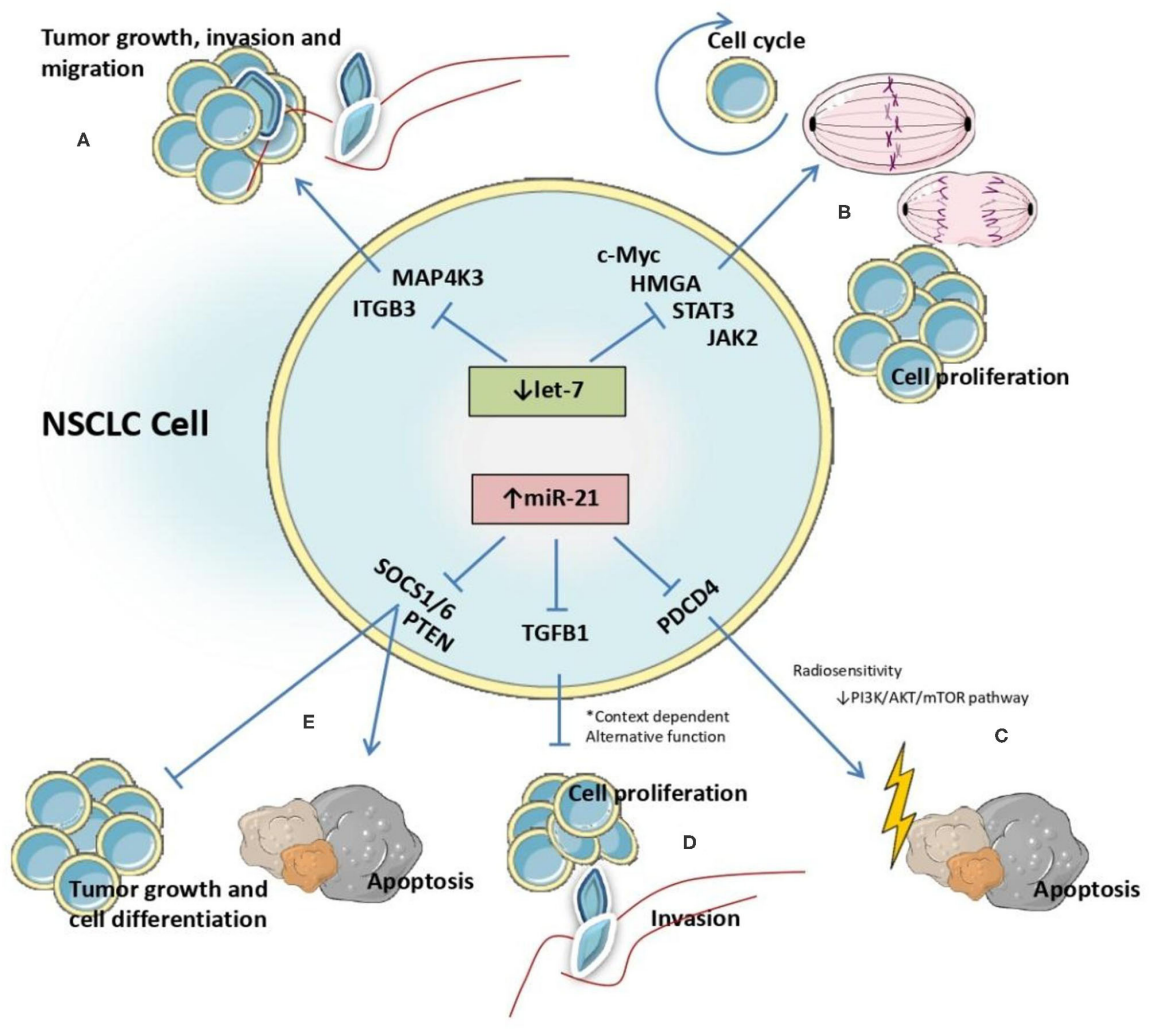
Identified interactions between let-7 and miR-21 microRNAs and target genes affecting molecular processes involved in NSCLC malignant transformation.

The impact of the anomalous expression of these two microRNAs is reflected in the cluttering of different molecular pathway, by targeting genes encoding proteins essential in these pathways. In NSCLC cell lines, miR-21 is reported to directly target phosphatase and tensin homolog (PTEN) a tumor suppressor gene (73), with effects on cell differentiation, tumor growth, apoptosis and cell proliferation (74). Also, it was reported that miR-21 targets transforming growth factor b-induced gene (TGFB1), which encodes the same protein with recognized functions in cell proliferation. Specifically, in A549 cells TGFB1 overexpression reduces cell proliferation and overexpression of miR-21 decreases TGFB1 levels, thus promoting aberrant cell proliferation and highlighting the function of miR-21 as an oncomiR in NSCLC (75). Also, there is solid evidence that miR-21 regulates radiosensitivity in lung cancer cells, and the mechanism reported by Jiang et al. indicate the interference with PI3K/AKT/mTOR signaling by inhibiting the transcription of pathway programmed cell death 4 (PDCD4) gene (76) (Figure 6).

The role of let-7 family members as tumor suppressors in NSCLC cells was evaluated in several publications that focused on the mechanism by which this molecule interacts with other genes. In SKMES-1 cells, the effect of let-7c as inhibitor of cellular growth, invasion and migration was demonstrated via downregulation of integrin beta-3 (ITGB3) and mitogen-activated protein kinase kinase kinase kinase 3 (MAP4K3). Let-7c reduces cell motility by binding to the 3'-UTR region of these genes and promoting their degradation (77).

In conclusion, our meta-analysis confirmed the predictive value of both miR-21 and let-7 for the outcome of patients with NSCLC. Besides, this investigation revealed that the impact of smoking is reflected in the survival rates of these patients, smoking patients having similar outcomes in both low expression/high expression groups for miR-21. One interesting finding refers to the fact that the accuracy of these molecules as predictors increases with the proportion of women in the sample. Future studies to evaluate the prognostic significance of other microRNAs may offer a complete understanding of the mechanisms of action of microRNAs in NSCLC and therefore would provide the basis to establish a miRNA/gene lung cancer panel that would help in assessing the outcome of NSCLC patients and therefore choose the right treatment course according to the molecular mechanisms affected reflected from the customized miRNA/gene panel. Given the evidence that miR-21 and let-7 expression remain statistically significant in assessing the outcome of NSCLC patients disregarding the type of biological sample used, these microRNAs appear to be valuable molecules to be taken into consideration for the set up of a miRNA/gene NSCLC cancer panel for prognostic.

## Conclusion

The present study revealed the applicability and stability of miR-21 and let-7 as prognostic biomarkers in NSCLC, disregarding the sample type—tissue sample or liquid biopsy. Our results highlight the importance of these two transcripts in lung cancer biology, particularly NSCLC, by interfering with crucial pathways associated with induction of apoptosis, cell cycle and cell proliferation. All these findings can represent valuable information for the management of NSCLC patients, with therapeutic significance, as this can count as a stepping stone for further studies focused on a better management of these patients.

## Data Availability Statement

Publicly available datasets were analyzed in this study. This data can be found here: https://pubmed.ncbi.nlm.nih.gov/–the meta-analysis was made by searching Pubmed database for relevant articles that were included in the analysis.

## Author Contributions

CP-B and LM: conceptualization, methodology, and writing. SP and RC: software and formal analysis. DG and MF: review and editing. IB-N: supervision and project administration. All authors contributed to the article and approved the submitted version.

## Conflict of Interest

The authors declare that the research was conducted in the absence of any commercial or financial relationships that could be construed as a potential conflict of interest.
