# Supplementary figures and images for: The Clinical Utility of miR-21 and let-7 in Non-small Cell Lung Cancer (NSCLC). A Systematic Review and Meta-Analysis
Source: Front Oncol. 2020 Oct 19;10:516850. doi: 10.3389/fonc.2020.516850 (PMC7604406; doi:10.3389/fonc.2020.516850)

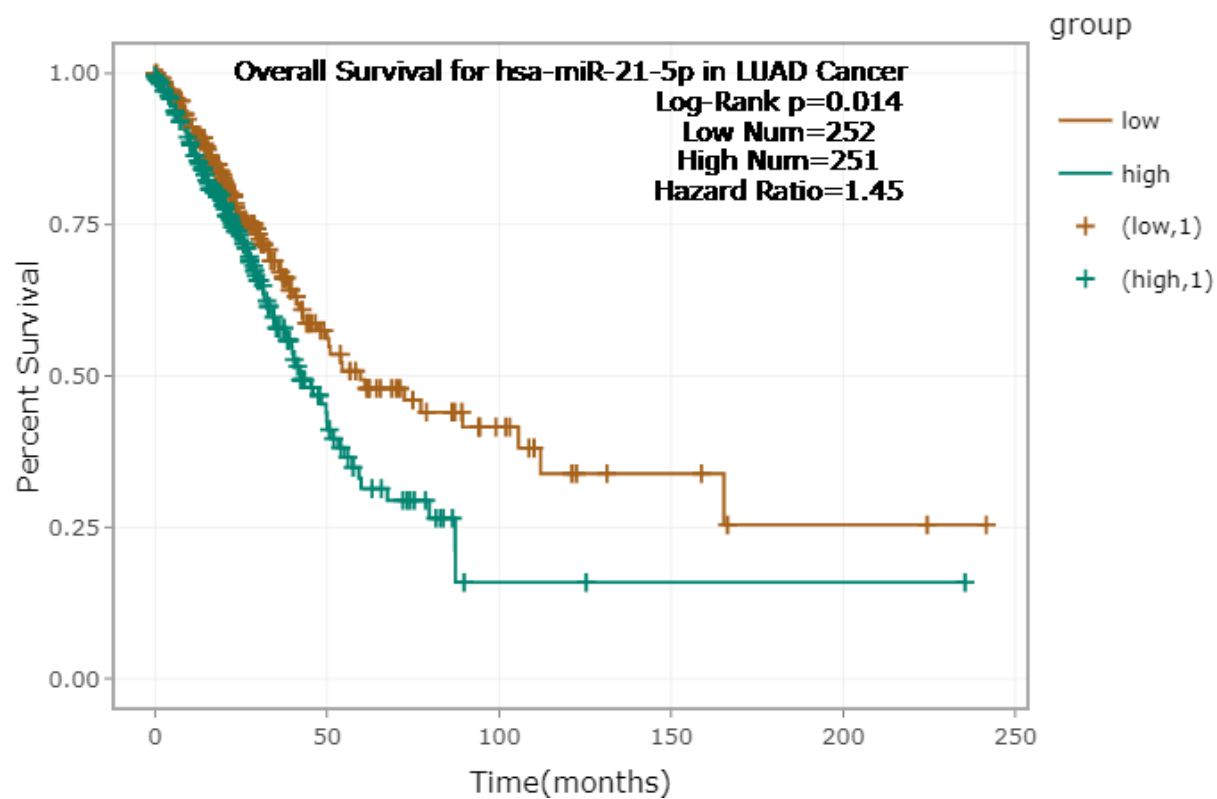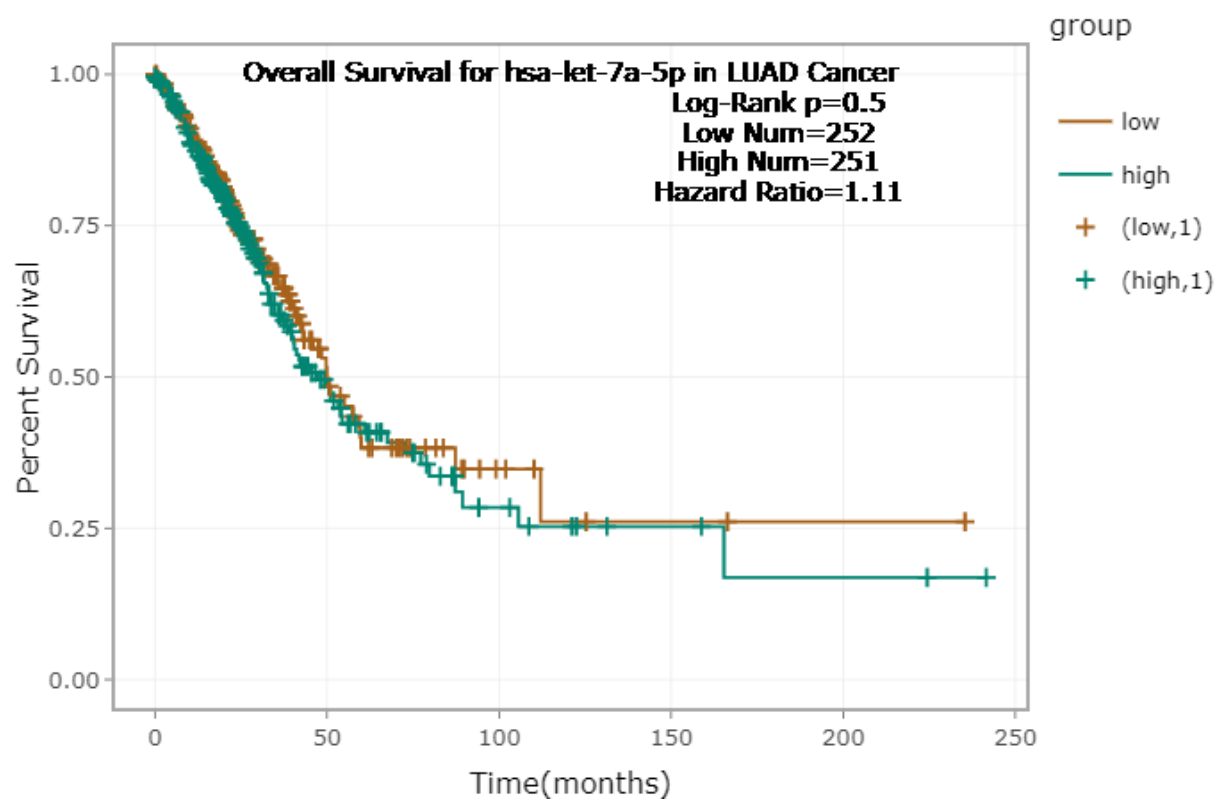

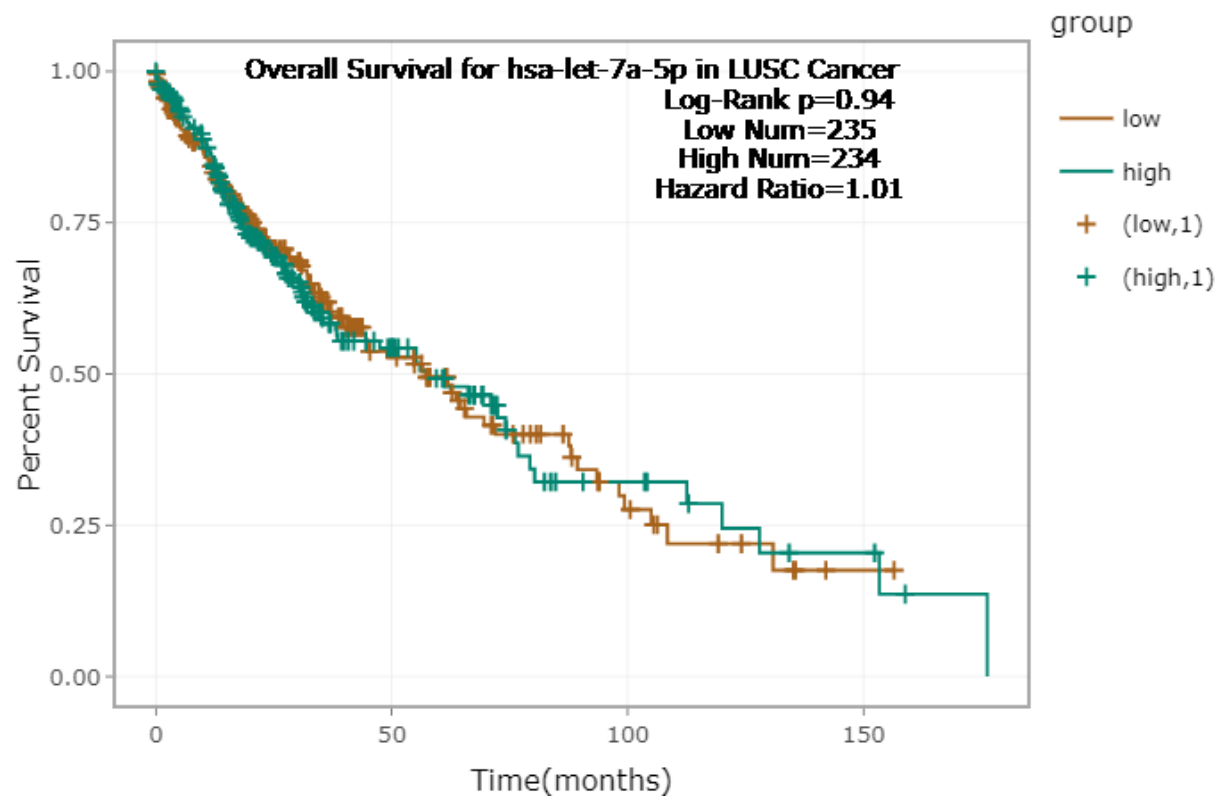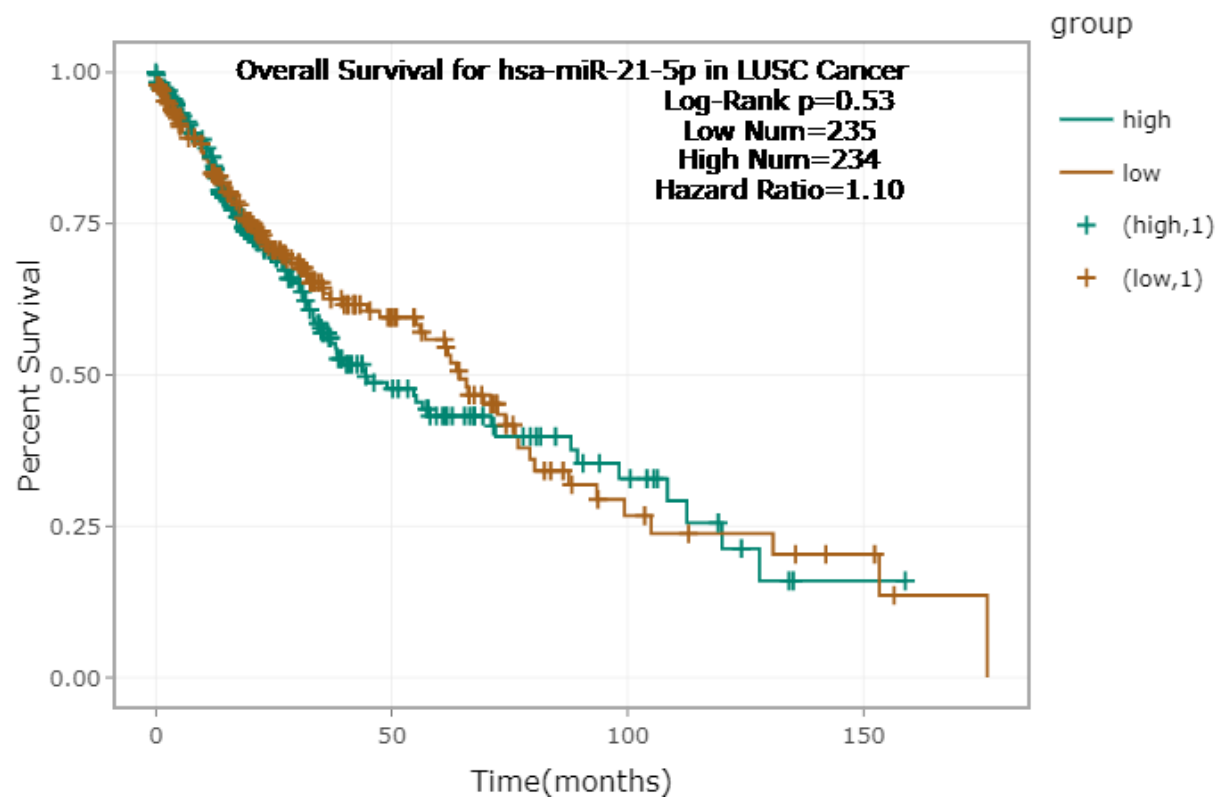

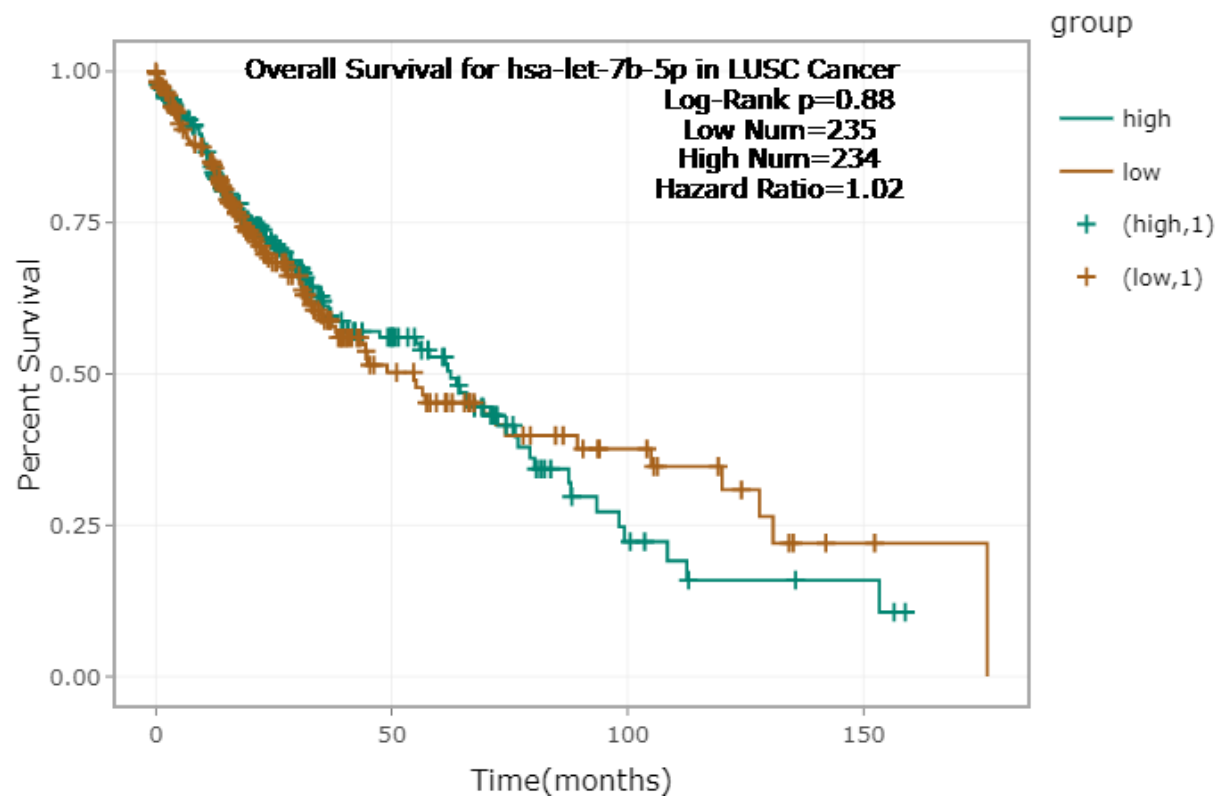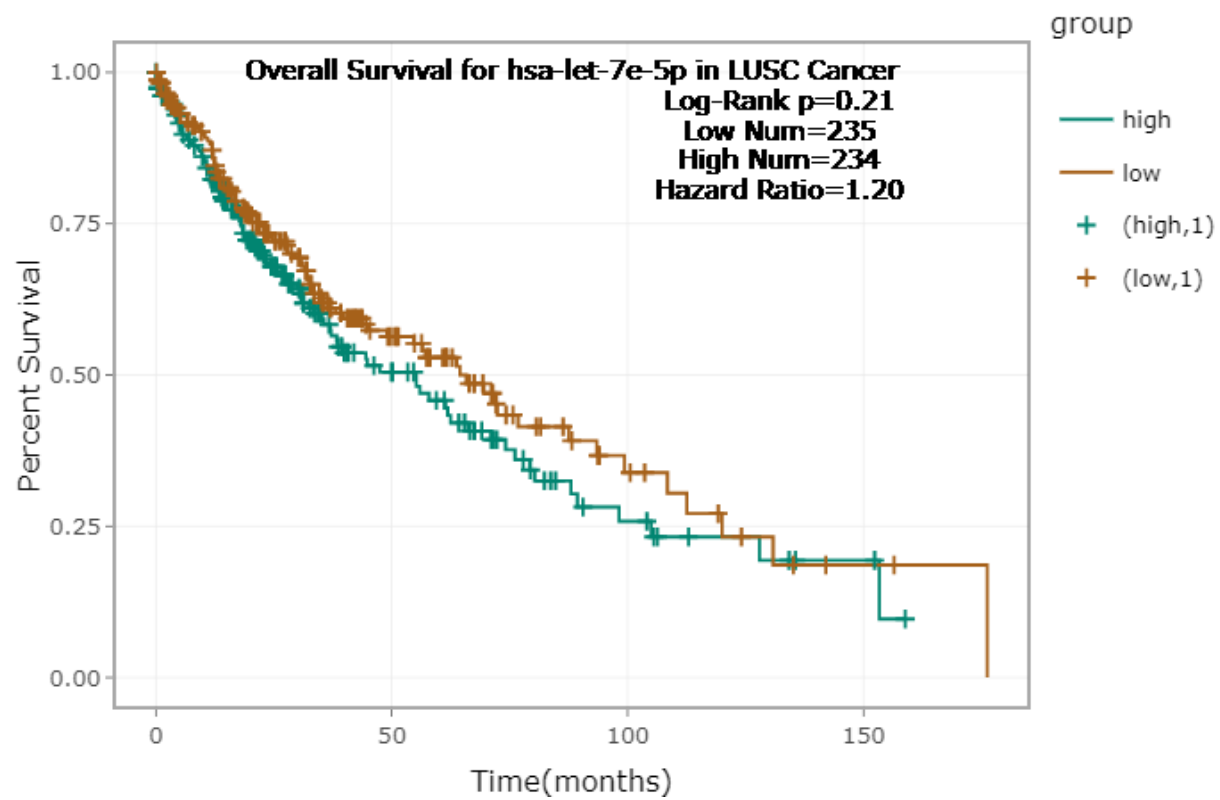

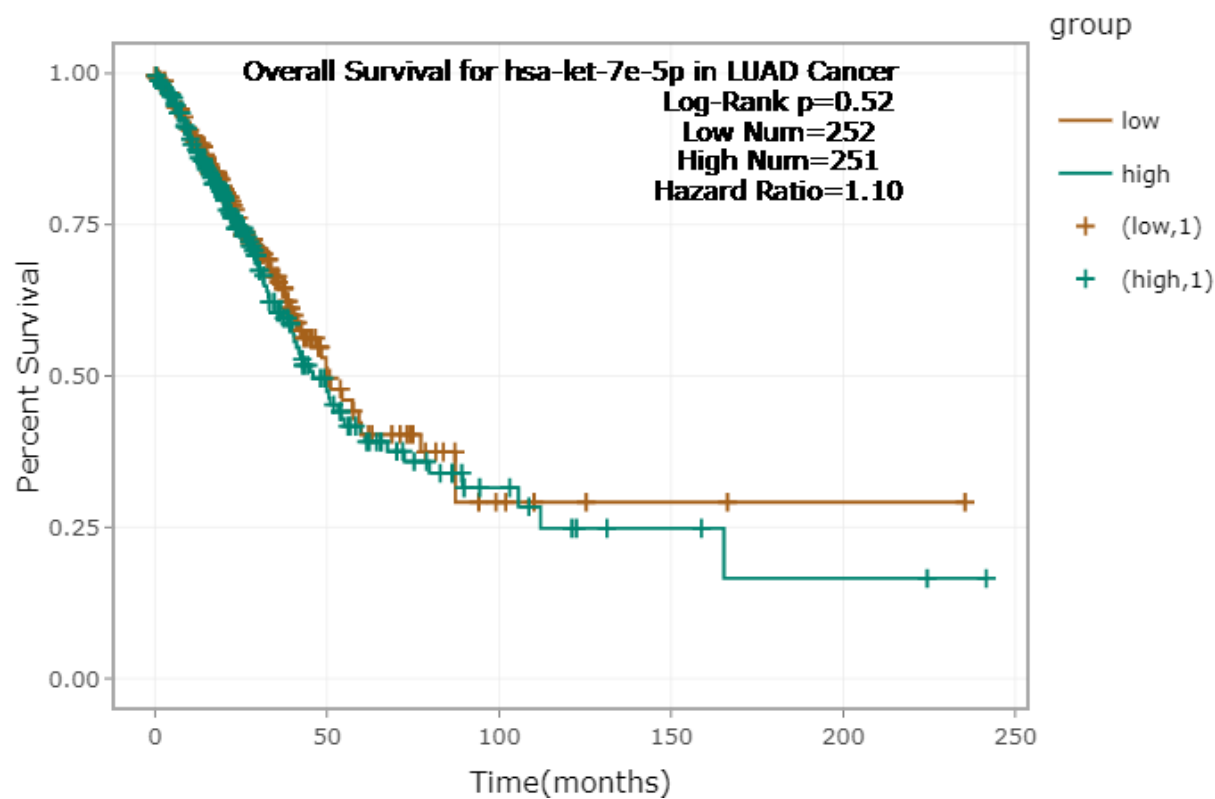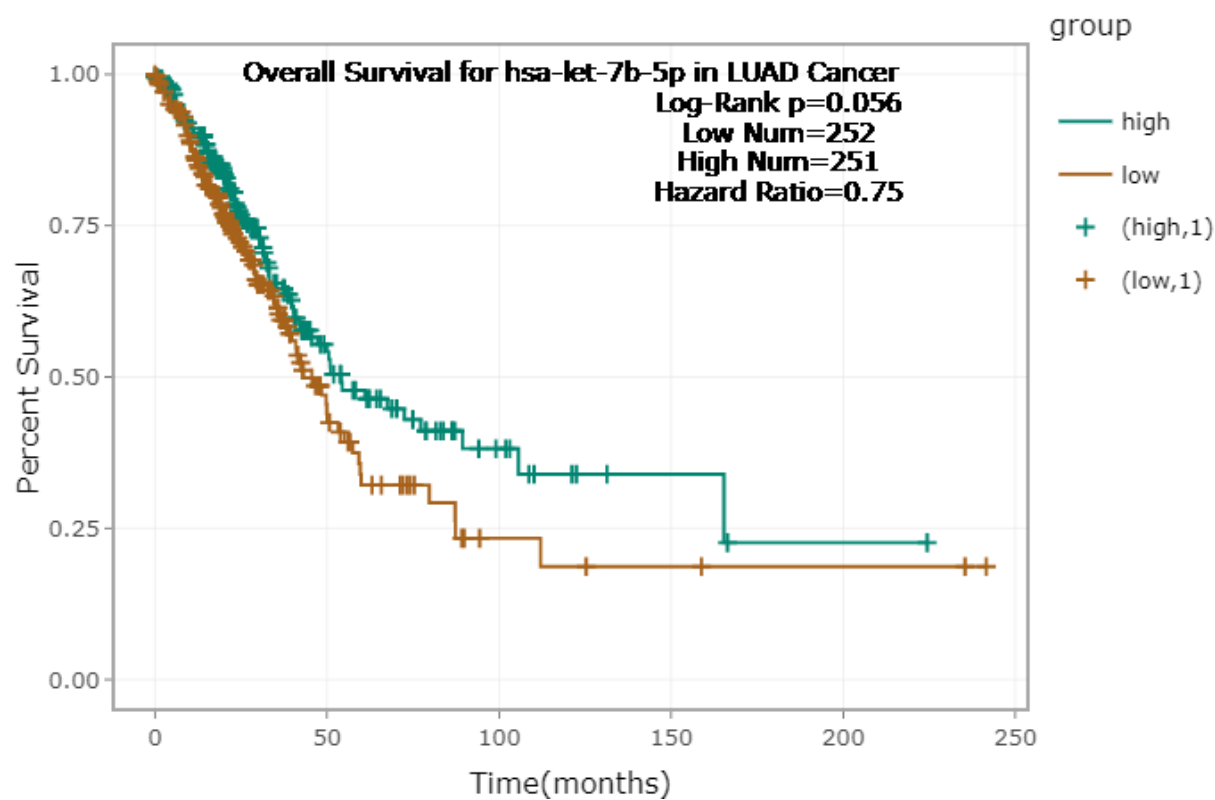

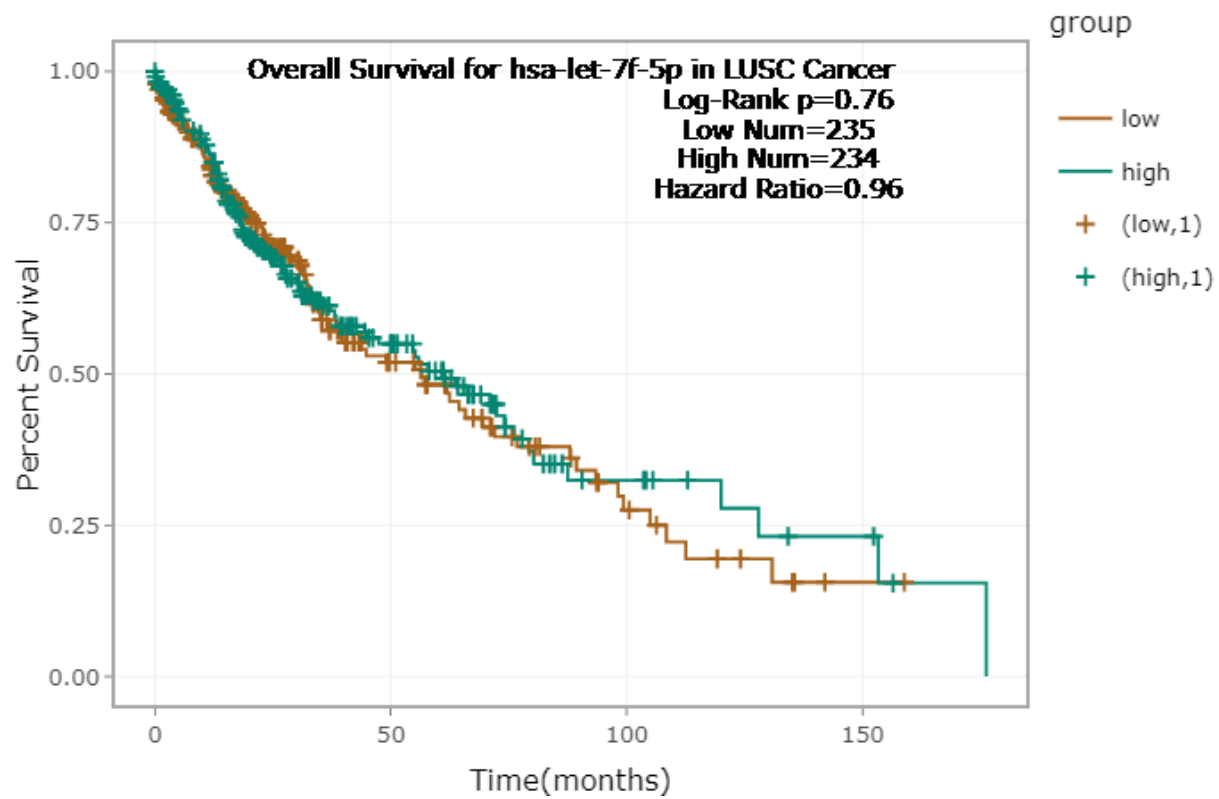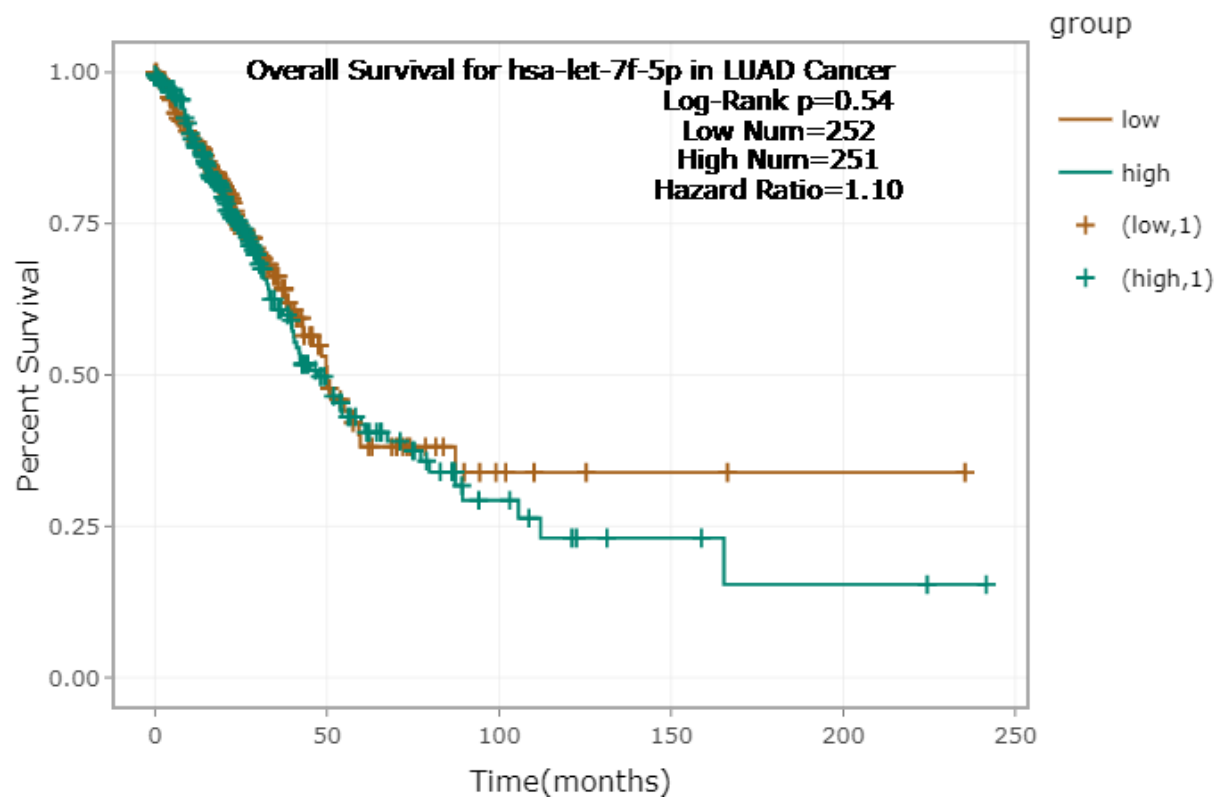

Supplement: Supplementary file 1 [file Data_Sheet_1.PDF]
